# Barriers and facilitators impacting access to sexual and reproductive health services for women with intellectual disability

**DOI:** 10.3389/fpsyt.2026.1795423

**Published:** 2026-07-16

**Authors:** Hiwot Temesgen Andualem, Susan A. Bartels, Tewodros Seyoum, Bilen Mekonnen Araya, Beata Batorowicz, Heather M. Aldersey

**Affiliations:** 1 School of Rehabilitation Therapy, Queen’s University, Kingston, ON, Canada; 2 Department of Public Health Sciences, Queen's University, Kingston, ON, Canada; 3 University of Gondar, School of Midwifery, Gondar, Ethiopia; 4 Faculty of Health Sciences, Western University (UWO), London, Canada

**Keywords:** access, community based rehabilitation (CBR), intellectual disability, sexual and reproductive health, women

## Abstract

**Background:**

In most parts of the world, people with disabilities are deprived of education, employment, health, and social and political rights. Moreover, women with disabilities can be subjected to double discrimination because of their gender and are more likely to experience physical, mental, and sexual abuse. Because of the nature of their disability, women with intellectual disability in particular face stigma and discrimination in accessing sexual and reproductive healthcare, which in turn affects their decision-making abilities regarding sexuality. In Ethiopia, due to a lack of resources and limited awareness, women with intellectual disabilities have difficulties accessing sexual and reproductive healthcare to meet their needs. We, therefore, aimed to explore the barriers and facilitators impacting access to sexual and reproductive health services in Gondar, Ethiopia.

**Methods:**

The study employed a qualitative descriptive approach to explore the existing barriers and facilitators impacting access to sexual and reproductive health services, aiming to inform how to improve service provision for women with intellectual disabilities in Gondar, Ethiopia. Using a semi-structured interview guide, we conducted interviews in Amharic with 17 purposively selected women with intellectual disability, aged 15-49, in Gondar, Ethiopia. After the interviews were transcribed, we conducted a thematic analysis using both inductive and deductive approaches in NVivo 14.

**Results:**

The analysis of the study resulted in six themes reflecting on different barriers and facilitators impacting Sexual and reproductive healthcare access: sexual and reproductive health education, contraception, prevention of sexual abuse, prevention of sexually transmitted infections, pregnancy and parenthood, and the roles of community-based rehabilitation impacting access to Sexual and reproductive health services.

**Conclusions:**

Study findings showed the barriers and facilitators impacting access to sexual and reproductive health services for women with intellectual disability, based on firsthand information and experiences of these women embedded in structural and sociocultural factors. The study’s findings could guide future researchers, healthcare policymakers and healthcare practitioners to design culturally appropriate and disability inclusive interventions promoting sexual and reproductive healthcare access and improved outcomes among women and girls with intellectual disability.

## Introduction

Access to quality sexual and reproductive health (SRH) services is essential to human well-being (1). In 2006, a comprehensive definition of sexual and reproductive health was developed by the World Health Organization (WHO). It is a state of complete physical, mental and social well-being and not just the absence of disease in one’s reproductive system and its functions and processes, including one’s ability to have fulfilling sex with the right to decide with whom, when and how often to have sexual encounters (2). Throughout life, sexual health is a vital and changing component of overall health. A person’s sexual health is influenced by determinants not only at individual levels but also at social levels, such as relationships, the community’s social environment, and the broader societal context (3). To promote SRH, it is necessary to reinforce education, health, and transportation services to guarantee that healthcare is accessible (4). The provision of SRH services relies on key factors, including efficient health infrastructure, integration with other services, the availability of essential supplies such as contraceptives and medications, and skilled healthcare providers who deliver prompt, courteous, and affordable care (4). Although SRH services are essential, typically marginalized populations, including people with disabilities, often lack access to them.

Women with disabilities, comprising about 19% of the world’s female population, experience double discrimination due to their gender and disability, making them more marginalized in society (5). In addition, women with disabilities are disproportionately more vulnerable to physical, mental and sexual abuse and face stigmatization around marriage and starting a family of their own as compared to women without disabilities (6). Notably, women with ID are among those who face such stigma and discrimination because of the nature of their disability (7), leading to limited research regarding factors affecting their access to SRH services. People with ID are sometimes forced to limit their engagement in SRH activities because they are thought to be asexual or childlike in relationships (8, 9). They may also be viewed as unsuitable parents or partners for marriage or other sexual relationships (8, 9). Due to the failure of health systems to prioritize their SRH and rights, people with ID face barriers to accessing care, resources, education, and information regarding SRH and are at greater risk for gender-based violence and abuse (8).

Access to SRH services, which is critical to achieving good health outcomes (10), is affected by numerous components of an individual’s life, including biological, psychological, social, emotional, physical, and spiritual aspects (11). Inadequate inclusion of people with disabilities in health sector policies; lack of knowledge about their SRH needs; misconception and poor attitudes of healthcare service providers and society; lack of social support (12); communicative and attitudinal barriers (13); lack of trained providers; inaccessible facilities; lack of transportation; high costs of care; unnecessary referrals; and risk factors like being low-income or experiencing violence (14) are among the barriers to accessing SRH services for people with disabilities.

An estimated 17.6% of the Ethiopian population lives with a disability (15), and among these, 95% live in poverty, leading to a lack of access to essential services (16). Article 41 (17) of the Constitution of the Federal Democratic Republic of Ethiopia states that “The State shall, within available means, allocate resources to provide rehabilitation and assistance to the physically and mentally disabled, the aged, and to children who are left without parents or guardians.” Moreover, Ethiopia is one of the 166 countries that have ratified the Convention on the Rights of Persons with Disabilities (CRPD) and are required to submit reports on how the convention is being implemented in the country. Given this, women with ID in Ethiopia have the right to access different kinds of services in various aspects of their lives, including sexual and reproductive healthcare. In Ethiopia, SRH access is highly influenced by social norms and cultural values reflecting stigma and misconceptions toward sexuality and SRH needs of women with ID, financial constraints and health care system limitations (18, 19). Moreover, the consideration of sexuality topics as taboo and sensitive has led to limited discussion on the SRH needs of women with ID. Even though the Ethiopian government is improving the expansion of SRH services, gaps still remain in inclusivity and access to SRH services for women with disabilities (20).

This study explores the existing barriers and facilitators impacting access to SRH services to improve service provision for women with ID in Gondar, Ethiopia. Specifically, it aims to answer the following research question: What barriers and facilitators impact access to SRH services for women with ID in Gondar, Ethiopia? With an emphasis on promoting inclusive treatment and reducing health disparities, this study contributes to empirical evidence by addressing key determinants, outcomes, and service gaps affecting people with ID.

## Methods

### Study design

We chose a qualitative descriptive approach as described by Sandelowski (21) to explore the barriers and facilitators impacting access to SRH services for women with ID. A qualitative descriptive approach is particularly relevant for guiding practitioners and policymakers in describing a given situation to inform practice or policy changes (21), which was our study objective. SRH among women with ID hasn’t been a significant focus of previous studies, and there is limited information about the existing situation in Ethiopia. Choosing a qualitative descriptive approach over other qualitative approaches (such as Ethnography, which focuses on thick description, Grounded theory, which focuses on development of a theory or Phenomenology, which focuses on interpretation of an experience).

### Study setting

This study took place in North Gondar, one of the cities in the Amhara region of Northern Ethiopia and 750 km Northwest of Addis Ababa, the capital city of Ethiopia. According to an Ethiopian Statistical Services report of the projected population of Ethiopia in 2025, Gondar’s total population is 532, 663, the female population being 271, 605 (22). There is no statistical information available on SRH demographics for the city. The study was conducted in partnership with the University of Gondar’s Community-Based Rehabilitation (UoG-CBR) Program, which was established in 2005 to provide rehabilitation services for people with disabilities. The program offers a range of rehabilitation and community inclusion-related services for more than 1500 people with disabilities in North Gondar, including women with ID.

### Study participants and recruitment

We used purposive sampling with a maximum variation strategy to select participants with varying socio-demographic characteristics (**Table 1**). Inclusion criteria included being a woman with ID who is within the reproductive age range (15–49), being a beneficiary of the UoG-CBR Program, speaking Amharic, and willingness to participate. Women with mild or moderate ID were selected over severe ID to ensure adequate communication for study participation. In the absence of formal diagnostic records, CBR workers assisted in identifying eligible participants based on program records. Seventeen women with ID were interviewed for the study. The concept of information power determined the final number of participants (23), given the narrow scope of the study’s aim and the quality of the dialogue we had with participants.

**Table 1:** Participants’ demographic information (N = 17).

Characteristics	Category	Number (%)
Age range in years	18-24	8 (47.1)
	25-30	6 (35.3)
	31-40	3 (17.6)
Marital status	Single	10 (58.8)
	Married	4 (23.5)
	In a relationship	2 (11.8)
	Divorced	2 (11.8)
Number of children	0	11 (64.7)
	1-2	4 (23.5)
	3-5	2 (11.8)
Level of education	No formal education	6 (35.3)
	Grades 1-4	6 (35.3)
	Grades 5-8	4 (23.5)
	Grades 9-10	1 (5.9)
Employment status	Unemployed	11 (64.7)
	Employed	6 (35.3)
Religion	Muslim	4 (23.5)
	Christian	13 (76.5)

### Data collection

We obtained ethical clearance from Queen’s University Health Sciences Research Ethics Board (HSREB), reference ID 6035345, and the University of Gondar Ethical Board. A UoG’s CBR worker, who had access to the program’s beneficiaries’ contact information, contacted the women, explained the study, and asked for their willingness to meet the first author (HT) in person for further explanation. HT met any interested women at a time and place convenient for them. During the meetings, she explained the research objective, obtained verbal consent, and offered three options: proceed with the next steps the same day, schedule another appointment if they were willing to participate, or decline to participate in the study. All participants agreed to proceed to the following steps on the same day. Participants were compensated with a moderate amount of money (400 Birr) for their time in participating in the interviews.

Before the interviews, we read and explained the consent forms to the women and conducted a screening to determine their capacity to consent to the interviews, as recommended by previous research conducted with people with ID (24, 25). The screening questions were: 1. Please tell me, in your own words, what this study is about? 2. What will you be doing if you take part in this study? 3. What are the risks of being in this study? 4. When I say taking part is entirely voluntary, what does that mean to you? 5. When I say that your answers will be kept confidential, what does that mean to you? and 6. What can you do if you start the study but don’t want to finish it? (24). Women who answered all of the above screening questions appropriately proceeded to the main interview. The interviews were conducted by HT in Amharic using semi-structured interview guides and lasted 30–60 minutes. During the data collection, one participant disclosed a sexual abuse that occurred years ago. We offered to provide referrals for emotional, legal, and medical support, but she declined, noting that the issue had already been dealt with legally.

### Data analysis

The interviews were transcribed and analyzed in Amharic to minimize the loss of meaning. During the translation, conducted by HT, we involved another Amharic-speaking colleague to validate the translations. A thematic analysis by Braun and Clarke was conducted, combining both inductive and deductive approaches (26). First, we familiarized ourselves with the data by repeatedly reading transcripts and notes from data collection and listening to interview audio recordings. Afterwards, we generated initial codes that might potentially be relevant to answering the research questions. Next, we aligned similar codes under predetermined themes developed based on the dimensions of healthcare access (27, 28). Subsequently, themes and sub-themes were checked for coherence with the codes to ensure they adequately answered the research questions. NVivo 14 was used for data analysis.

## Results

For this study, 17 women with mild-to-moderate ID and diverse demographic characteristics were interviewed (**Table 1**). The participants come from diverse sociodemographic backgrounds and possess a range of experiences regarding SRH. These varying perspectives significantly shape their understanding and experiences in these areas. Results are presented under SRH topics as they relate to the six dimensions of access (**Table 2**). We employed Penchansky and Thomas’s modified theory of access as an analytical framework. They identified five dimensions central to health services, which Saurman (19) later improved by incorporating awareness as an additional dimension, emphasizing the importance of communicating about existing healthcare services to promote effective access for the given population.

**Table 2:** Dimensions of access (28).

Dimensions of access	Definition	Dimension components and examples
Accessibility	Location	An accessible service is within reasonable proximity to the consumer in terms of time and distance. In this study’s context, Access to SRH services was influenced by physical proximity and women with IDs’ reliance on others for transportation.
Availability	Supply and demand	An available service has sufficient services and resources to meet the volume and needs of the consumers and communities served. In this study’s context, a range of SRH services, including contraceptive methods (e.g., condoms, pills, IUDs, injections), was reported as available, though consistency of provision varied.
Acceptability	Consumer perception	An acceptable service responds to the provider and consumer’s attitude regarding the service’s characteristics and social or cultural concerns. In this study’s context, social and cultural attitudes toward disability and sexuality, along with stigma and perceived judgment, influenced women with ID’s willingness to seek SRH services.
Affordability	Financial and incidental costs	Affordable services examine the direct costs for the service provider and the consumer. In this study, indirect costs, such as transportation and SRH-related expenses (e.g., menstrual products and other services), were discussed as limiting consistent access to SRH services.
Adequacy (Accommodation)	Organization	Adequate service is well organized to accept clients, and clients can use the services. Considerations of adequacy include hours of operation (after-hour services), referral or appointment systems, and facility structures (wheelchair access). In this study, service organizations and provision, including varying levels of tailored support for women with ID, influencing their ability to understand and use SRH services were discussed.
Awareness	Communication and information	A service maintains awareness through effective communication and information strategies with relevant users (clinicians, patients, and the broader community), including consideration of context and health literacy. In this context, the lack of consistent and targeted SRH information, delivered in simple and understandable formats, limiting women with ID’s knowledge and their ability to make informed decisions were discussed.

### Sexual and reproductive health education

Participants were asked where they get information about sex-related topics. Except for a few women who had not attended school, most women had access to SRH-related topics through formal education. Some of the subjects they were taught included sex, contraception, and menstrual hygiene. Mebrat said,

“Our biology book talked about sex and stuff.”

One participant stated that her school provided SRH education only for female students. Emebet said,

“They gave us advice to practice abstinence.”

Some other women stated that SRH information or education was unavailable in their schools.

The women also discussed their access to SRH information through the media. Although a few women indicated that they got no information about SRH from the media, some women stated that they learned about sexual intercourse, STIs and sexual abuse from television dramas. Emebet added,

“When we listen to FM radio, they said that we should have one partner and that diseases can be transmitted sexually.”

One woman said that she did not have access to the media because she could not afford it. Sofia said,

“We don’t have television or radio; we live in a poor household.”

The interviewed women stated that family was another source of SRH information, such as menstrual hygiene. Some of them said that they learned about sex from their parents, and a few women mentioned that they learned about sex from their spouses.

Moreover, several women said SRH information was available in healthcare centers. They said that healthcare workers mainly taught them about different contraception methods. Other sources of SRH information included their friends, parents, spouses, and community members. Some women indicated that they could also be a source of SRH education for others. For example, Abeba stated,

“I advise my friends to learn from my experiences. I tell them to focus on their education. I tell them not to have sex with a guy unless they are tested. I also tell them to get contraceptive injections, not to get pregnant. As long as we are women, it is mandatory to be careful. I tell them not to end up like me.”

Aside from all the SRH information sources discussed by participants, a few women also revealed that they have no idea what sex is and have never had a sexual encounter before. Furthermore, many of the women highlighted that they do not discuss SRH topics with anyone; in fact, some of them described sex as an indecent topic to discuss. One participant stated that even though she does not know much about the matter, she wants to learn about it in the future.

### Contraception

Women were asked about their awareness of contraception methods. Some women indicated that they had never heard of or used any contraception method. One participant stated that she had heard of contraceptives but did not know where to get them. On the other hand, most of the participants said that they knew about different types of contraception methods and listed condoms, IUDs, contraceptive pills, and contraceptive injections as the most prominent. Among these, contraception injections were used by most of the interviewed women. One participant claimed that injections are suitable methods for labor work. Abeba stated,

“The IUD [Intrauterine Device] (pointing to her stomach) is not suitable for work. See, I wash clothes for a living, and the loop is uncomfortable to work. What is comfortable for me is the injection (pointing to her arm).”

Most women were not using any contraception at the time of the interview. Participants gave different reasons for this, including a plan to give birth, considering contraceptives as a cause for infertility, and deeming the use of contraceptives as a sin. Mebrat said,

“I don’t want to use contraception. It is forbidden in Christianity. It is a sin.”

On the contrary, a few other women said that the use of contraception helped them avoid unwanted pregnancy, made them happy and enabled them to enjoy sex without the fear of getting pregnant. Abeba added.

“Using contraception makes you enjoy sex and makes you happy. If I didn’t use contraception, I wouldn’t be happy and might get pregnant again.”

When women were asked about how they accessed contraceptives, most of them mentioned that they got contraceptives from government-based healthcare centers where contraceptives are provided free of charge. One woman said that she buys contraceptives from private pharmacies and that she does not get any financial support to purchase them. Another woman stated that she knows what contraceptives are, but has no information about where to get or buy them.

The women also discussed where they obtained information about contraception. They stated their parents, friends, and neighbors as their sources of information about different types of contraception. Hirut said,

“Sometimes my friends talk about how they got contraceptive implants not to get pregnant.”

Hanna added,

“My mother sometimes, in the middle of a conversation, mentions how she got the contraceptive injection.”

The women also discussed the decision to use contraception. Multiple women noted that their family members supported them in their decision to use contraceptives. They said that sometimes, their family members, friends and even doctors made decisions for them on the type of contraception they should use. A few other women stated that they made such decisions for themselves.

### Prevention of sexual abuse

When asked about whether they know what sexual abuse is, the majority of women stated that they do not have any information about what it is. On the other hand, very few women indicated that they knew what sexual abuse meant and tried to give a few examples, such as rape, intimidation, and early marriage. Emebet stated,

“In school, they told us that child marriage is a bad practice.”

Abeba added,

“Attack is, for example, when someone comes from the back while you are walking on the street and kidnaps you and rape you.”

These women also mentioned that school, the media, and other people’s experiences were their sources of information about sexual abuse. Among the interviewed participants, one woman in particular revealed that she was a victim of sexual abuse. She indicated that she received the proper help and that the perpetrator was imprisoned for his crime.

### Prevention of sexually transmitted infections

Most interviewed women indicated that they know what STIs are, even though they only mentioned HIV/AIDS when asked to list some. A few women said that they had never heard of HIV/AIDS and any other sexually transmitted infections. In a discussion regarding their awareness of HIV/AIDS, some participants tried to explain their understanding of HIV/AIDS testing, transmission, and prevention. As only one participant mentioned that she did not know about testing, most participants said they were aware of HIV/AIDS testing and its benefits. They mentioned the freedom to live a healthy life, a chance for happiness, an opportunity to take care of oneself and receive support from others as benefits of HIV/AIDS testing. Hirut stated.

“I got tested for HIV, and I was free; I was so excited. Other couples I knew also got tested and were free as well, they seemed very happy with the result.”

Even one woman stated that she regularly gets tested every three months. Abeba said,

“I get tested for HIV/AIDS every three months to know about my health status. I even get my child get tested.”

Multiple participants also mentioned that they receive support from family members to get tested for HIV/AIDS. One woman in particular said that she and her boyfriend got tested together.

Moreover, most of the women indicated that they are aware of ways HIV/AIDS is transmitted, even listing a few. Hagere said,

“HIV is transmitted by sexual intercourse and needles and sharp objects.”

Furthermore, most participants showed their awareness of HIV/AIDS prevention methods by discussing a few. Addisie mentioned,

“Well, to avoid getting HIV, the main thing is reserving oneself from having sex. Otherwise, one should marry the best for them and be contained in a marriage.”

The women also revealed that they get information about STIs, specifically about HIV/AIDS, from their parents, other peoples’ experiences and discussions with friends.

Some of the participants described that healthcare centers are one of the places where HIV/AIDS testing and information are available. Some women stated that they had access to free HIV/AIDS testing at their local healthcare center. They said that healthcare workers helped them get tested for HIV/AIDS and taught them about HIV/AIDS transmission and prevention methods. A few other women reported receiving the same services from a government hospital. Moreover, a few women stated that information about HIV/AIDS was also available in their schools. Although most participants knew about HIV/AIDS and some were regularly tested, the majority indicated they had never accessed any STI testing themselves, including for HIV/AIDS.

Interviewees also discussed the cost of getting tested for STIs, including HIV/AIDS. Among the women who had done a test for HIV/AIDS, all of them noted that they had not paid for the service. In Ethiopia, government healthcare centers and hospitals provide free HIV/AIDS counselling, testing and diagnosis services to encourage the population to get tested and prevent the spread of the virus.

### Pregnancy and parenthood

The interviewed women discussed their awareness of pregnancy and child raising. Except for a few women who said that they knew nothing about pregnancy, most women stated that they understood the concept of pregnancy and other issues related to child-raising. Some women discussed how to raise a child and noted essential aspects of taking care of a child, including getting their child tested for HIV/AIDS. Addisie said,

“My friends and I talk about raising a child. A mother should not abandon her kids; she should raise them well. I would rather beg for money to raise my children than abandon them.”

Regarding their encounters accessing pregnancy follow-ups and maternal healthcare services, most women who had been pregnant received their antenatal care in a government hospital free of charge. The government of Ethiopia encourages the provision of free antenatal care to promote healthy pregnancy and reduce the deaths of mothers and children as a result of a lack of medical care. One of the women said that she got to see the development of the fetus during those follow-ups. Abeba stated,

“I went to the hospital for a pregnancy follow-up. They used to follow up my blood pressure and gave me medications for it. They informed me about the status and position of the fetus. They told me what to do and what not to do for the baby’s health.”

Moreover, this participant mentioned that a nurse at the hospital informed her about pregnancy care and the prevention of HIV/AIDS. Another woman said that she had a pregnancy follow-up in a private healthcare setting, paying for the services. Multiple women indicated that they had given birth at home without medical assistance; one of them said that the healthcare center was far from her house, making it difficult to travel and deliver her baby. She also added that she never had a medical follow-up throughout her pregnancy due to the long distance from the healthcare center. Girum said,

“The hospital is very far. We even have to cross a river to get there.”

Most women said they had received support from spouses, parents, siblings, and other family members during their pregnancy and while raising their children. Abeba said,

“My parents were happy when I got pregnant. They get excited about his future. The house feels empty for them when he is not around.”

Mebrat added,

“My parents encouraged me to go to the clinic for a pregnancy check-up.”

A few women also said they got pregnancy and child-raising tips from their parents and neighbors—some of the advice they got included breastfeeding and sunbathing their newborns. Abeba shared,

“My neighbors advised me how to breastfeed my newborn. They also advised me on how to sunbathe and massage my child so as not to cause him muscle weakness.”

Mebrat indicated,

“My mother advised me on what and how to feed my baby. She told me to give them milk and solid foods when they get bigger. She told me that they will start crawling after one year old.”

Moreover, several women who had not yet been pregnant reported plans to have children in the future. On the other hand, several others stated they have no plans to become parents.

### Community-based rehabilitation services

At our study location, the CBR program at the University of Gondar provides multisectoral support for women with ID and people with disabilities in general. Study participants shared their experiences with the University of Gondar’s CBR program as it related to their access to SRH services. A few women stated they did not know the CBR program existed, even after it was explained to them. Other participants stated that the CBR program provided food support, including biscuits for children and flour, butter, and drinks for women who had given birth. Abeba stated,

“When I gave birth, CBR workers drove to my house and gave me food and drinks; I will never forget that.”

One woman added that the CBR also provided her with financial support during pregnancy and after to help her raise her child.

Participants also discussed the different lessons they got from CBR workers, including lessons about contraception, raising a child, HIV/AIDS prevention and keeping menstrual hygiene. Moreover, CBR arranged a 1-to-5 grouping method for women to get together, learn from, and support each other on various SRH issues. However, one woman indicated that all the CBR support stopped during the acute phase of the COVID-19 pandemic. Multiple women expressed their interest in the continuation of the CBR help. They also expressed a need for more support, especially in clothing, housing, schooling, food and drinks, healthcare services, including SRH, and support in raising children.

## Discussion

This study explored the barriers and facilitators impacting access to SRH services for women with ID. The findings illustrated that barriers such as stigma, societal misconceptions, lack of access to SRH services and facilitators such as community and healthcare service providers support impacting their experiences with diverse SRH aspects, including SRH education, contraception, prevention of sexual abuse and STIs, and experiences of parenthood and roles of CBR in promoting SRH access. Additionally, the findings of the study emphasized the complex interplay of gender, disability, and sociocultural factors that significantly influence the experiences of women with ID in seeking SRH services. These intersecting aspects not only shape their individual challenges but also affect their overall access to vital healthcare resources and support systems. Considering this, we used Penchansky and Thomas’s modified theory of access as an analytical framework. Penchansky and Thomas’s (27) theory of access identifies five dimensions of access as central to health services. These dimensions are accessibility, availability, acceptability, affordability, and accommodation (27). The modified theory of access by Saurman (28) incorporates awareness as an additional dimension. In this improved theory of access, awareness emphasizes the importance of communicating about existing healthcare services to promote adequate access to healthcare for the given population (28).

In Ethiopia, in addition to the lack of healthcare infrastructure to provide SRH services, a lack of awareness within the population about the existing services and whether people with disabilities need the services also contributes to poor access to SRH services by women with ID (19). Our findings showed the experience of women with ID in accessing SRH services in relation to SRH education, contraception, prevention of sexual abuse, prevention of sexually transmitted infections, and pregnancy and parenthood. These findings highlight the importance of tackling attitudinal and structural barriers to deliver holistic and inclusive strategies to support people with ID across different life aspects.

People with ID need to have access to information about SRH topics while exploring their sexuality. The reliance on informal resources such as friends, spouses, parents, the media (Television), and community members for SRH information was indicated by participants. Other studies have also mentioned similar sources of SRH information (7, 29, 30). The findings suggest that women with ID have some access to SRH information, particularly in areas such as sex, contraception, menstrual hygiene, and sexually transmitted infections. However, the scope of this information appears limited, indicating gaps in comprehensive sexuality education. Existing literature emphasizes the importance of including additional components such as self-protection skills, sexual consent, and reproductive physiology to better support the needs of women with ID (7, 30, 31). Although it has been evident that having SRH knowledge has a broader benefit in reducing people with IDs’ vulnerability to sexual abuse and enhances their ability to make better decisions about their sexuality and sexual well-being (32–34), our study participants explained that the SRH information they acquired benefited them in areas of contraception use and prevention of STIs. Historically, there have been disparities in providing SRH services for women with ID, including sex education (32, 35–37), even though they have the same sexual desires and sexual education requirements as women without disabilities (33). Many of our study participants revealed that SRH issues are not up for discussion and considered the topic to be “indecent”. In addition to the topic of sex being mostly taboo in Ethiopian society (38), people with disabilities are not encouraged to share their sexual experiences with others. This resulted in women with ID often relying on informal sources of information and education, shaping their understandings and experiences of SRH issues.

Contraception was one of the important topics discussed by study participants. Access to different contraception methods is associated with benefits for SRH. Prevention of unwanted pregnancy and the ability to experience pleasurable sex are commonly identified advantages, aligning with findings from previous studies on women with ID (7, 39, 40). Researchers have indicated that it is crucial to evaluate how and what types of contraceptives women with ID use since their limited communication and lack of access to contraception information highly affects their decision-making (41). Our study participants listed condoms, IUDs, contraceptive pills, and contraception injections as the contraception methods that are available and accessible to them. Some participants in our study indicated that the decision to choose their methods of contraception rests with them, while others reported that friends, family members, and even doctors made these decisions on their behalf. This dynamic contradicts the principles outlined in Article 25 of the CRPD (42), which affirms the rights of people with disabilities to informed consent, dignity, and autonomy in making independent decisions about the healthcare services they are receiving, including SRH services, that significantly impact their well-being. Those women who made decisions for themselves relied on a simple personal preference, to avoid unwanted pregnancies, looking at other people’s experiences with contraceptives and based on their suitability for their work. On the contrary, several studies indicated various factors in contraceptive selection, such as menstrual-related behavioral concerns, hygiene concerns (43–46), and consideration of their vulnerability to sexual abuse (41, 47). Some of our study participants stated that others make contraceptive use decisions for them, as similarly found in previous studies. It is common for family members and healthcare professionals to make contraception selections for women with ID with the perception that they cannot make such decisions for themselves (45). Even though our study participants had not experienced it, evidence shows that women with ID experience involuntary sterilization forced by family members and healthcare professionals (48–50), violating Article 10 (2) of the United Nations Office of the High Commissioner for Human Rights strictly prohibiting both the sterilization of and the performance of an abortion on, a woman with disabilities without her prior informed consent (51). Women with ID need to be provided with thorough and easily understandable information regarding the various kinds of contraceptive methods and their usage to support them in making informed choices. While communication strategies may need to be tailored, it is crucial to provide clear and appropriate SRH information and to ensure their autonomy and privacy, thereby upholding their rights to accessible information and to free and informed consent, as stated in Articles 21 and 25 of the CRPD (42).

The majority of women in our study said they had no idea what sexual abuse or violence was, but this does not mean they had never been the victim of sexual abuse in their lives, because they are less likely to report it (52, 53). Numerous studies show that women with ID are at a higher risk of being exposed to sexual abuse than women without disabilities (7, 54–57). Since our participants listed only rape, intimidation, and early marriage as examples of sexual abuse, more could have been done to equip them with knowledge about various other types of sexual abuse. Numerous studies have pointed out the importance of sexual abuse knowledge, including consent (7, 55), how and where to report abuse (53), and self-defense strategies (58) to prevent individuals from sexual abuse. These findings highlight that women with ID should be presented with the information and resources to understand what sexual abuse is and protect themselves, as well as be able to report incidents without prejudice.

As indicated in different studies, little information is available regarding STIs among women with ID (7, 55, 59), even though the existing studies show that women with ID are more vulnerable to STIs than people without disabilities (31, 60–62). Similarly, HIV/AIDS emerges as the only sexually transmitted infection identified, suggesting that available STI information for women with ID is limited. Knowledge of HIV/AIDS transmission and prevention is evident, with government hospitals and healthcare centers serving as key sources of free information and testing services. This is the result of the HIV/AIDS National Strategic Plan of Ethiopia, 2021-2025. This plan was prepared by the Federal HIV/AIDS Prevention and Control Office (FHAPCO) in collaboration with the Ethiopian Ministry of Health and other government sectors. The plan covers numerous topics concerning HIV/AIDS testing, diagnosis, prevention and transmission among different populations. The plan encourages and supports free HIV/AIDS counselling and testing, free provision of HIV/AIDS information in healthcare centers and hospitals, provision of free Anti-Retroviral medications, and free condom distribution, all to reduce HIV/AIDS infections and related mortality rate in Ethiopia (63). Our study participants reported that they are benefiting from this government plan in terms of receiving free HIV/AIDS services.

Pregnancy and parenthood emerge as important and relevant issues. Some individuals have direct experience with pregnancy and parenthood, while others have observed these experiences in others. Historically, women with ID faced and are still facing stigma toward getting pregnant, raising children and starting their own family (64), which in turn made them less likely to receive appropriate prenatal care (64–67). A few studies in a South African context (68) explored how societal and caregivers’ attitudes and concerns regarding the parenting capabilities of people with ID, as well as the implications for them becoming parents and caring for their children. These pervasive beliefs contribute to the stigmatization of individuals with ID, significantly impacting their ability to form intimate relationships and pursue parenthood. Negative experiences with healthcare and social systems can inflict deep emotional trauma, eroding their confidence and overall sense of well-being. Mothers in this demographic frequently confront a troubling combination of dismissiveness and discrimination from both professionals and society at large, which exacerbates their feelings of isolation and alienation. Such experiences not only diminish their support networks but also hinder their active engagement in their communities (65, 69, 70). However, our study participants experienced this differently. They explained how their families and communities supported them during their pregnancies and afterward as they raised their children. In Ethiopia, a child’s birth is considered a big step in forming a family. The culturally and religiously conservative Ethiopian community, most of the time, shows support and care for pregnant women and their newborns. As some people oppose women with ID having children, many others support it, believing that children will grow up and support their mothers (71). Moreover, free prenatal care is available through healthcare centers and government hospitals. In an effort to reduce maternal and infant death, pregnancy and childbirth-related healthcare services in government healthcare facilities are free in Ethiopia (72, 73). As explained by one of our participants who was forced to give birth at home, infrastructural barriers such as lack of transportation and inaccessibility of healthcare services in Ethiopia are reasons for women not to receive prenatal care and give birth in healthcare facilities (72, 73). Even though our study participants’ experiences were different, evidence shows that women with ID often lack accessible prenatal information, emotional and social support (70, 74). Our study only reflects the situation in a specific location in Ethiopia and may not accurately represent the situation nationwide. SRH is still a demanding topic which requires the attention of health care policy makers, mainly in tailoring services to meet the needs of women with ID in Ethiopia.

Community-based rehabilitation (CBR) is a strategy to ensure that people with disabilities have equal rights and opportunities to other members of society, with equal access to healthcare services, education, skills training, employment, family life, social mobility, and political empowerment (75). One of the organizations in Gondar that provides rehabilitation services for people with disabilities is the University of Gondar’s Community-Based Rehabilitation Program (UoG-CBR), established in 2005. The program provides different kinds of services for more than 795 people with disabilities, including women with ID (76). Our study participants described the different types of support they have received from the UoG-CBR program. Schindler & Aldersey (42) explored the potential for CBR to support SRH service provision, sexual abuse prevention, and HIV/AIDS programming, services and education and found that there are relatively limited programs that offer such services, with potential for other CBR programs to implement them in the future (77). Our participants mentioned a few services they received from the CBR program in the areas of contraception, HIV/AIDS, child-raising and menstrual hygiene. This shows that the CBR program is improving its SRH services for women with ID. Participants also expressed their desire for service provision to continue and improve in the future. As CBR programs are closest to the community, they should be able to identify inequalities and injustices in SRH care provision and develop resources and solutions to address them.

### Limitations

This study has certain limitations. This study was conducted in Gondar, with a unique societal and cultural context from other parts of Ethiopia. Therefore, it is inappropriate to generalize the results of this study to the vast Ethiopian population, which has diverse cultural contexts. SRH is not a widely and openly discussed subject in the area where the interviews were conducted. The participants showed some reservations in opening up about their SRH experiences. Moreover, due to the nature of the women’s disabilities, it took extra effort to establish communication and gain their trust to proceed with the interviews. We made an effort to ensure a friendly atmosphere that would encourage participants to feel comfortable discussing the interview questions. Nevertheless, it is still possible that some participants were not comfortable sharing certain aspects of their SRH experiences. The political unrest and security instability in the study setting also posed challenges. We were forced to delay several interviews for the safety of participants and interviewers.

## Conclusion

Our study showed the barriers and facilitators women with mild and moderate ID face in accessing SRH services. It involved capturing the women’s experiences rather than having caregivers or healthcare professionals speak on their behalf. Understanding the SRH service needs of women with ID is crucial in designing and providing tailored SRH services that will enable them to lead healthy and fulfilling lives. Our study has potential implications regarding research, policy, and practice.

Although this study was conducted with a limited number of participants and at select study sites, it demonstrates that SRH among women with ID is a broad topic and represents a significant area for future research, both within the same study location and across other regions of Ethiopia. Future researchers can draw insights from our findings on the challenges and opportunities associated with this population, potentially leading to improved outcomes. Moreover, our study advances the field by going beyond commonly reported barriers and facilitators to access to SRH services, offering a perspective on African sociocultural norms and how they influence women with IDs’ experiences of accessing SRH services. Ethiopian healthcare policy has gaps in addressing the SRH needs of people with disabilities, ranging from the lack of available demographic data on this population to the protection of the healthcare rights of people with disabilities. Policymakers could benefit from the information directly from the experience of women with ID themselves, since future policies should be designed to address their challenges and concerns.

These study findings suggest that future interventions to promote access to SRH services for women with ID would be successful if they involve the women themselves and the social support around them. Peer and social support for these women has great potential, as they have already established strong relationships with family members, friends, and the community, paving the way for practitioners to build on and strengthen them. This study highlights the critical need for healthcare policies that are both inclusive of individuals with disabilities and culturally sensitive. By prioritizing these aspects, we can create an environment that ensures equitable access to SRH services for women with ID.

## Data Availability

The datasets presented in this article are not readily available to protect the confidentiality and anonymity of participants. Requests to access the datasets should be directed to the corresponding author.
